# In Vitro and In Vivo Antiviral Activity of Nylidrin by Targeting the Hemagglutinin 2-Mediated Membrane Fusion of Influenza A Virus

**DOI:** 10.3390/v12050581

**Published:** 2020-05-25

**Authors:** Yejin Jang, Jin Soo Shin, Joo-Youn Lee, Heegwon Shin, Sang Jick Kim, Meehyein Kim

**Affiliations:** 1 Infectious Diseases Therapeutic Research Center, Korea Research Institute of Chemical Technology (KRICT), Daejeon 34114, Korea; erythro1@krict.re.kr (Y.J.); jsshin@krict.re.kr (J.S.S.); leejy@krict.re.kr (J.-Y.L.); 2 Department of Chemistry, Korea Advanced Institute of Science and Technology (KAIST), Daejeon 34141, Korea; heegwon.shin@kaist.ac.kr; 3 Synthetic Biology and Bioengineering Research Center, Korea Research Institute of Bioscience and Biotechnology (KRIBB), Daejeon 34141, Korea; sjick@kribb.re.kr; 4 Graduate School of New Drug Discovery and Development, Chungnam National University, Daejeon 34134, Korea

**Keywords:** influenza A virus, hemagglutinin 2, fusion inhibitor, nylidrin, β2-adrenergic receptor

## Abstract

Influenza A virus, one of the major human respiratory pathogens, is responsible for annual seasonal endemics and unpredictable periodic pandemics. Despite the clinical availability of vaccines and antivirals, the antigenic diversity and drug resistance of this virus makes it a persistent threat to public health, underlying the need for the development of novel antivirals. In a cell culture-based high-throughput screen, a β2-adrenergic receptor agonist, nylidrin, was identified as an antiviral compound against influenza A virus. The molecule was effective against multiple isolates of subtype H1N1, but had limited activity against subtype H3N2, depending on the strain. By examining the antiviral activity of its chemical analogues, we found that ifenprodil and clenbuterol also had reliable inhibitory effects against A/H1N1 strains. Field-based pharmacophore modeling with comparisons of active and inactive compounds revealed the importance of positive and negative electrostatic patterns of phenyl aminoethanol derivatives. Time-of-addition experiments and visualization of the intracellular localization of nucleoprotein NP demonstrated that an early step of the virus life cycle was suppressed by nylidrin. Ultimately, we discovered that nylidrin targets hemagglutinin 2 (HA2)-mediated membrane fusion by blocking conformational change of HA at acidic pH. In a mouse model, preincubation of a mouse-adapted influenza A virus (H1N1) with nylidrin completely blocked intranasal viral infection. The present study suggests that nylidrin could provide a core chemical skeleton for the development of a direct-acting inhibitor of influenza A virus entry.

## 1. Introduction

Influenza A, belonging to the family *Orthomyxoviridae*, causes contagious human respiratory diseases in pandemics and seasonal or zoonotic epidemics. Its genome is composed of eight negative-stranded RNA segments, each of which is encapsidated with nucleoprotein (NP) through interactions with subunits of the viral polymerase (polymerase basic protein 1 [PB1], PB2, and polymerase acidic protein [PA]) to generate viral ribonucleoproteins (vRNPs) [1,2]. The viral lipid bilayer envelope bears spikes consisting of major glycoproteins, hemagglutinin (HA) and neuraminidase (NA), which determine the serologic and antigenic properties of viral strains, along with a relatively small population of matrix protein 2 (M2). Underneath the membrane, the matrix protein 1 (M1) layer selectively packages eight different vRNPs arranged in the “1 + 7” pattern [3]. Viral infectivity depends on the function of homotrimeric HA at the initial step of the viral life cycle, making this surface protein attractive as an antiviral target. Each HA monomer is composed of two disulfide-linked polypeptides, HA1 and HA2, produced by proteolytic cleavage of the HA0 precursor. The globular head domain of HA, accounting for most parts of HA1, recognizes α-2,3- or α-2,6-linked sialic acid receptors on host cells prior to viral internalization into the endosome [4,5]. Binding of the virus to the receptor triggers endocytosis. At the acidic environment of endosomes (pH 5.0–6.0), the M2 proton channel opens to facilitate proton transport into the viral interior, causing vRNPs to dissociate from the inner M1 layer, and HA undergoes structural rearrangements, resulting in exposure of the HA2 fusion peptide from a hidden hydrophobic pocket and its subsequent insertion into the endosomal membrane [6,7]. These simultaneous events create a route for the escape of vRNPs into the cytoplasm, followed by their migration to the nucleus, mediated by nuclear localization signals (NLSs) within NP [8], where viral RNA replication and transcription occurs.

There are three classes of clinically available antivirals targeting NA, M2, or PA [9,10]. NA inhibitors (NAIs) including oseltamivir, zanamivir, peramivir, and laninamivir block NA-mediated cleavage of sialic acids attached to HA during the very late stage of infection and thus interfere with the release of progeny viruses from infected cells. Adamantanes such as amantadine and rimantadine attenuate M2 proton channel activity, which is required for virus–endosome membrane fusion. The most recently developed drug is the PA endonuclease inhibitor, baloxavir marboxil, which prevents the cleavage of 5’ caps from host mRNAs during the “cap-snatching” process, enabling initiation of viral mRNA synthesis. Despite the high potency of these drugs against influenza A and/or B viruses, the emergence or global spread of drug-resistant mutants is an increasing public health concern, and such mutations have arisen even in the absence of drug pressure [11]. The majority of currently circulating H1N1 and H3N2 isolates are resistant to adamantanes due to amino acid mutations in M2 (e.g., S31N and V27A [12]). The H274Y mutation in NA of H1N1 and H5N1 and the E119V and R292K mutations in H3N2 NA are associated with dramatically diminished sensitivity to NAIs [13,14]. Moreover, the I38T mutation in PA in the 2009 pandemic H1N1 and seasonal H3N2 strains confers reduced susceptibility to baloxavir marboxil [15]. This widespread drug resistance highlights the need for the development of antivirals with an alternative mode of action to respond to possible pandemics caused by multi-drug-resistant viruses.

Previously, we have screened a chemical library of approximately 7000 small molecules by the cytopathic effect (CPE) reduction assay using fluorescein diacetate (FDA); hit compounds were those that allowed 80% of influenza A or B virus-infected cells to survive at a final concentration of 20 µM [16,17]. One of the hits, nylidrin showed antiviral activity against influenza A viruses, but not against influenza B viruses (Table 1). Also known as buphenine, nylidrin is a β2-adrenergic receptor (ADRB2 or B2AR) agonist classified as a vasodilator [18]. On the basis of broad-spectrum antiviral evaluation using its chemical derivatives, we found that two additional analogues, ifenprodil and clenbuterol, reliably inhibited H1N1 isolates of the influenza A virus. 

In the present study, we suggest that nylidrin suppresses the membrane fusion step by preventing the structural changes of HA that occur at low pH. Accordingly, preincubation of influenza virus with nylidrin completely blocked intranasal infection of mice. Thus, nylidrin represents a new class of anti-influenza agents that act by blocking the HA2 function. Our findings provide insights into further chemical modifications of the hit compounds that could lead to the development of a direct-acting antiviral against influenza A with a distinct and novel mode of action.

## 2. Materials and Methods 

### 2.1. Cells and Viruses

Madin–Darby canine kidney (MDCK), African green monkey kidney (Vero) E6, and A549 cells purchased from American Type Culture Collection (ATCC, Manassas, VA, USA) were cultured in minimum essential medium (MEM; Gibco/Invitrogen, Carlsbad, CA, USA), Dulbecco’s modified Eagle’s medium (DMEM; Gibco/Invitrogen), and RPMI-1640 medium (Gibco/Invitrogen), respectively, supplemented with 10% fetal bovine serum (FBS; Gibco/Invitrogen) at 37 °C. Influenza viruses A/Puerto Rico/8/1934 (H1N1; PR8), A/Hong Kong/8/1968 (H3N2; HK), and B/Lee/40 (Lee) were also obtained from ATCC, and A/California/7/2009 (H1N1), A/Brisbane/59/2007 (H1N1), A/Seoul/11/1988 (H3N2), A/Perth/17/2009 (H3N2), A/Brisbane/10/2007 (H3N2), A/Victoria/361/2011-like (H3N2), and B/Brisbane/60/2008 were provided by the Korea Centers for Disease Control and Prevention (KCDC, Chungcheongbuk-do, Republic of Korea) [19]. Viruses were amplified by infection of 10-day-old embryonated chicken eggs or MDCK cells in the presence of 2 µg/mL TPCK-trypsin (Sigma-Aldrich, St. Louis, MO, USA) for three days. Allantoic fluids or cell culture supernatants were harvested by centrifugation at 3000 rpm for 10 min, and aliquots were stored at −70 °C before use. Viral titers were determined by plaque assay. Mouse-adapted PR8 virus (maPR8) was prepared as described previously [19].

### 2.2. Compounds and Cytopathic Effect (CPE) Reduction Assay

The test compounds nylidrin hydrochloride (~95%), ifenprodil (+)–tartrate salt (≥98%), labetalol hydrochloride (≥98%), ritodrine hydrochloride (99.6%), fenoterol hydrobromide (≥98%), eliprodil (≥98%), clenbuterol hydrochloride (≥95%), and bambuterol hydrochloride (≥98%) were purchased from Sigma-Aldrich. As control compounds, the M2 inhibitor amantadine hydrochloride (AMT; ≥98%) and the polymerase inhibitor ribavirin (RBV; ≥98%) were also from Sigma-Aldrich. The NA inhibitor oseltamivir carboxylate (OSV-C; ≥98%) was acquired from United States Biological (Swampscott, MA, USA).

Cell-based antiviral assays were performed as previously described [19,20]. Briefly, MDCK cells were seeded on 96-well plates (3 × 10^4^ cells per well) and grown overnight. The cells were mock-infected or infected with individual influenza strains at a multiplicity of infection (MOI) of 0.001 to 0.005 for 1 h at 33 °C or 35 °C. After the removal of unadsorbed virus, test and control compounds were serially 3-fold diluted from 100 to 0.01 µM (total ten concentration points) in serum-free MEM with 2 µg/mL tosyl phenylalanyl chloromethyl ketone (TPCK)-treated trypsin and used to treat the cells. To synchronize the reading time point, at three days post-infection (p.i.), the plates were incubated at the following temperatures: 33 °C for A/California/7/2009, A/Brisbane/59/2007, A/Perth/17/2009, A/Brisbane/10/2007, A/Victoria/361/2011-like, and B/Brisbane/60/2008); and 35 °C for PR8, HK, Lee, and A/Seoul/11/1988. The half-maximal cytotoxic concentration (CC_50_) and half-maximal effective concentration (EC_50_) were determined by measuring cell viability using 3-(4,5-dimethylthiazol-2-yl)-2,5-diphenyltetrazoliumbromide (MTT).

### 2.3. Field-Based Pharmacophore Modeling

For the development of a ligand-based pharmacophore model, seven compounds were selected from the phenyl aminoethanol derivatives used in the CPE assay. On the basis of antiviral efficacy against A/H1N1 strains, nylidrin, ifenprodil, and clenbuterol were defined as active compounds as they have selectivity indices over 20, whereas labetalol, ritodrine, fenoterol, and eliprodil were designated inactive or less active ones. Conformation of the compounds was generated to a maximum of 200 conformations using the Conformation Hunt module in Forge v.10.3 (Cresset, Litlington, UK). The 3D common field point pattern for three active compounds were generated using FieldTemplater in Forge v.10.3 by the default setting. Four different molecular field points such as positive and negative electrostatic, shape, and hydrophobicity were calculated by using Cresset’s eXtended Electron Distribution (XED) force field [21]. The top 3D field alignment model was selected where the resulting templates included three active compounds. To compare with the 3D common field point pattern from the inactive compounds, the same procedure was also employed using FieldTemplater. The top 3D field alignment model was selected where the resulting templates included the four compounds’ inactive compounds.

### 2.4. Immunoassays

To detect viral proteins, western blot analysis was performed as described previously [16]. MDCK cells grown to 100% confluence in 6-well plates were infected with PR8 virus at an MOI of 0.001 for 24 h at 35 °C in the presence of nylidrin, OSV-C, or RBV. Viral proteins NP, HA, and M1 were detected by immunoblotting the appropriate antibodies: mouse anti-NP (cat no. 11675-T62; Sino Biological, Beijing, China), rabbit anti-HA2 (cat no. 86001-RM01; Sino Biological), and mouse anti-M1 (cat no. sc-57881; Santa Cruz Biotechnology, Dallas, TX, USA), respectively. Secondary antibodies were horseradish peroxidase (HRP)-conjugated goat anti-mouse or anti-rabbit IgG (Thermo Fisher Scientific, Waltham, MA, USA). Cellular β-actin was used as the loading control.

### 2.5. Plaque Assay

PR8-infected MDCK cells were mock-treated or treated with the individual compounds. After 24 h at 35 °C, culture supernatants were harvested to prepare 10-fold serial dilutions, which were used to infect naïve MDCK cells seeded in 48-well plates for 1 h. After PBS washing, they were incubated in overlay medium (MEM with 0.6% carboxymethylcellulose (CMC; Sigma-Aldrich) and 2 µg/mL TPCK-trypsin) at 33 °C for three days. Plaques were counted by crystal violet staining.

For time-of-addition experiments, MDCK cells in 48-well plates were infected with PR8 (MOI, 0.001) at 4 °C for 1 h in the presence or absence of nylidrin. After washing with PBS to remove chemicals and unadsorbed virus, nylidrin was administered at 1, 2, and 4 h p.i. (−)-Epigallocatechin gallate (EGCG; Sigma-Aldrich) was used as a control for blocking viral entry. Subsequently, all samples were washed with PBS at 5 h p.i., followed by incubation under the overlay medium for three days prior to crystal violet staining.

### 2.6. Confocal Microscopy

For fluorescence microscopy, PR8-infected MDCK cells were incubated for 5 h at 37 °C in the presence or absence of nylidrin. After fixation and permeabilization, viral NP was visualized using anti-NP antibody (cat no. sc-80481; Santa Cruz Biotechnology) and Alexa Fluor 488-conjugated goat anti-mouse IgG (Invitrogen); nuclear DNA was counterstained using 4´,6-diamidino-2-phenylindole (DAPI; Vector Laboratories, Burlingame, CA, USA). Images were captured on a Zeiss LSM 700 confocal microscope with analysis using the ZEN software (Carl Zeiss, Thornwood, NY, USA).

For investigating colocalization of viral NP with cellular Rab5 or Rab7, A549 cells were seeded in 4-well slides (4 × 10^4^ cells per well). On the next day, cells were transfected with 0.4 μg of pEGFP-Rab5 or pEGFP-Rab7 (a kind gift from Prof. C. Bucci at Università del Salento, Lecce, Italy) using lipofectamine 2000, according to the manufacturer’s instructions (Invitrogen). After 24 h, they were mock-infected or infected with PR8 at an MOI of 10 in the absence or presence of 100 μM nylidrin at 4 °C for 30 min and then at 37 °C for an additional 4 h. Viral NP protein was probed using the same primary antibody above-mentioned, but with Alexa Fluor 633-conjugated secondary antibody (Invitrogen).

### 2.7. Polykaryon Assay

Syncytia formation caused by influenza infection was evaluated as described previously [16,22]. Vero E6 cells were infected overnight at 37 °C with PR8 at an MOI of 1. After treatment with TPCK-trypsin (5 µg/mL) for 15 min, they were treated with either nylidrin (20 or 100 µM) or anti-HA2 antibody (5 µg/mL) for an additional 15 min. The samples were washed with 1 mM MgCl_2_ and 0.1 mM CaCl_2_ dissolved in PBS (PBS-CM) and incubated in acidic (pH 5.4) or neutral (pH 7.0) PBS-CM supplemented with nylidrin or the antibody for 15 min, followed by incubation for 3 h in DMEM with 10% FBS. Giemsa staining (Sigma-Aldrich) allowed the visualization of cell–cell fusion. Herein, a well-known neutralizing antibody for influenza A virus HA2, MEDI8852, was chosen as a control for the HA-mediated fusion assay [23]. In brief, the scFv genome sequence of MEDI8852 was synthesized and cloned into pDR-OriP-Fc1 for mammalian cell expression in a form of scFv-Fc [24]. The construct was transfected into HEK293E cells (ATCC), and protein G agarose (Merck Millipore, Darmstadt, Germany) was used for affinity purification of the expressed scFv-Fc.

### 2.8. Trypsin Protection Assay

Trypsin susceptibility of HA was tested at 37 °C as previously described with some modifications [22]. Influenza A virus PR8 (5.5 × 10^7^ PFU/mL) was incubated with increasing concentrations (0, 40, and 400 µM) of nylidrin for 20 min. Acidic pH (5.0 or 5.2) was achieved by adding predetermined amounts of 1 mM sodium acetate buffer (pH 4.8). Samples were incubated for 15 min, and the medium was neutralized (pH 7.0) by the addition of 0.5 M Tris-HCl (pH 9.0). Virus was digested with trypsin for 1 h, and the reaction was stopped by the addition of the SDS sample buffer. After 12% SDS-PAGE and electrotransfer, both HA1 and HA2 proteins were probed with rabbit anti-HA1 (cat no. 11684-T54; Sino Biological) and rabbit anti-HA2 antibodies (cat no. 86001-RM02; Sino Biological), respectively, followed by incubation with HRP-conjugated goat anti-rabbit secondary antibody as described above. 

### 2.9. In Vivo Study

Female BALB/c mice (6–7 weeks old; Orient Bio Inc., Gyeonggi-do, Republic Korea) were infected with mouse-adapted PR8 (maPR8). Five units of 50% mouse lethal dose (5 MLD_50_) of maPR8 was preincubated with nylidrin or DMSO as a control for 30 min at room temperature. Mice were intranasally challenged with maPR8 in the presence or absence of nylidrin at a dose of 10 mg/kg in a total volume of 50 μL. In another control group, oseltamivir phosphate (OSV-P [>98%], provided by Hanmi Pharmaceutical Co., Gyeonggi-di, Korea) was administered from days 0 to 5 p.i. at a dose of 10 mg/kg/day (b.i.d.) beginning 4 h before virus challenge. Body weight changes and mortality were measured every day for 15 days. Mice were sacrificed when they lost at least 30% of their body weight, in accordance with the ethics guidelines approved by the Institutional Animal Care and Use Committee (IACUC) of the Korea Research Institute of Chemical Technology (KRICT). The identification code of IACUC protocol is 2020-6D-03-01. The approval date of the committee was 12 March 2020. Kaplan–Meier survival curves were drawn using GraphPad Prism 6 (GraphPad Software, San Diego, CA, USA).

### 2.10. Statistical Analysis

Data are presented as mean ± standard error of the mean (SEM) of three independent experiments. Comparisons between groups were analyzed using an unpaired, two-tailed unpaired *t*-test or a two way AVONA with Dunnett’s multiple comparisons. *p*-values below 0.05 were considered to represent statistical significance.

## 3. Results

### 3.1. Anti-Influenza Viral Activity of Nylidrin and Its Analogues

In a screen of a chemical library consisting of 7000 small molecules [16,17], we identified nylidrin as a hit compound that inhibits influenza A viruses (PR8 and HK). At a concentration of 20 µM, nylidrin maintained 80% viability in MDCK cells infected with influenza A, but conferred no benefit in CPE-reduction following infection with an influenza B strain (Lee). To verify this finding, we performed antiviral and cytotoxicity assays at various concentrations of nylidrin with a high purity of about 95% (3-fold serial dilutions from 900 to 0.05 μM) using AMT, RBV, and OSV-C as the controls. The data showed that nylidrin had EC_50_ values of 7.2, 12.1, and >100 µM against the PR8, HK, and Lee strains, respectively, and a CC_50_ value of 549.2 µM in mock-infected MDCK cells, resulting in selectivity indices (SI) of 76.3 against PR8 and 45.4 against HK (Table 1).

Nylidrin (also known as buphenine) is a β2-adrenergic agonist [25]. On the basis of its structural and functional properties, we selected seven additional chemical analogues to investigate the relationship between chemical structure and antiviral activity (Supplementary Figure S1). In the CPE-based antiviral assay, ifenprodil was comparable to nylidrin, with EC_50_ values of 6.6 and 16.9 μM for PR8 and HK, respectively (Table 1). Clenbuterol was potently active only against PR8 with an EC_50_ value of 9.4 μM, whereas labetalol and eliprodil were only marginally effective against HK alone with EC_50_ values above 44.0 μM. In contrast, ritodrine, fenoterol, and bambuterol had no antiviral activity against any viral strains. In further experiments, we tested the inhibitory compounds, even partially including nylidrin, ifenprodil, labetalol, eliprodil, and clenbuterol, against additional influenza A and B viruses (Table 2). Nylidrin, ifenprodil, and clenbuterol were consistently effective against A/H1N1 strains, but their efficacy varied among the H3N2 viral strains, and none had antiviral activity in influenza B-infected cells. As expected, the remaining two compounds, labetalol and eliprodil, had little effect against any of these viral strains, except for A/Brisbane/59/2007 (H1N1), which was partially sensitive to eliprodil (EC_50_, 53.1 μM). These results indicate that nylidrin and its analogues, ifenprodil and clenbuterol, can reliably inhibit the infection of H1N1 strains of the influenza A virus at subtoxic concentrations.

### 3.2. Field-Based Pharmacophore Analysis of Phenyl Aminoethanol Compounds

**Figure 1:**
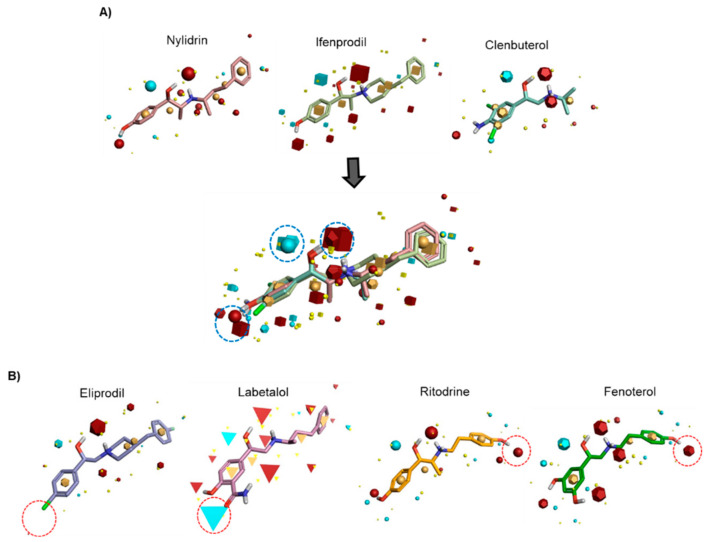
Identification of common pharmacophore active regions on the basis of field points from the ligand-based pharmacophore model. (**A**) Field-based template model of three active molecules, nylidrin, ifenprodil, and clenbuterol, and their merged image. Highly conserved electrostatic domains are highlighted in dotted blue circles. (**B**) Representation of four template compounds with little or no activity. Unfavorable electrostatic domains, distinct from the active compounds, are marked in dotted red circles. Field points are visualized as follows: blue, negative electrostatic potential; red, positive electrostatic potential; orange, hydrophobic; and yellow, van der Waals.

To identify the pharmacophore characteristics of these antiviral compounds, we performed ligand-based pharmacophore modeling (Figure 1). Upon merging the three active compounds, nylidrin, ifenprodil, and clenbuterol, on the basis of their hydrophilic and hydrophobic field points, we found one negative and two positive electrostatic regions around the phenyl aminoethanol center (Figure 1A). Interestingly, the two marginally active compounds, eliprodil and labetalol, against HK and A/Brisbane/59/2007 lost one of the positively charged fields adjacent to the central phenyl ring (Figure 1B). Even though the completely inactive compounds, ritodrine and fenoterol, had main electrostatic regions similar to those of the active compounds, they had an extra positive electrostatic potential at the amino terminal phenol ring, which may hinder their specific interaction with a target protein or provide an unfavorably charged region, disrupting an essential hydrophobic interaction. Together, these results indicate that conservation of the three electrostatic potential sites of the phenyl aminoethanol derivatives is critical for anti-influenza viral activity in cells.

### 3.3. Abnormal Accumulation of Nucleoprotein (NP) in the Cytoplasm by Nylidrin

**Figure 2:**
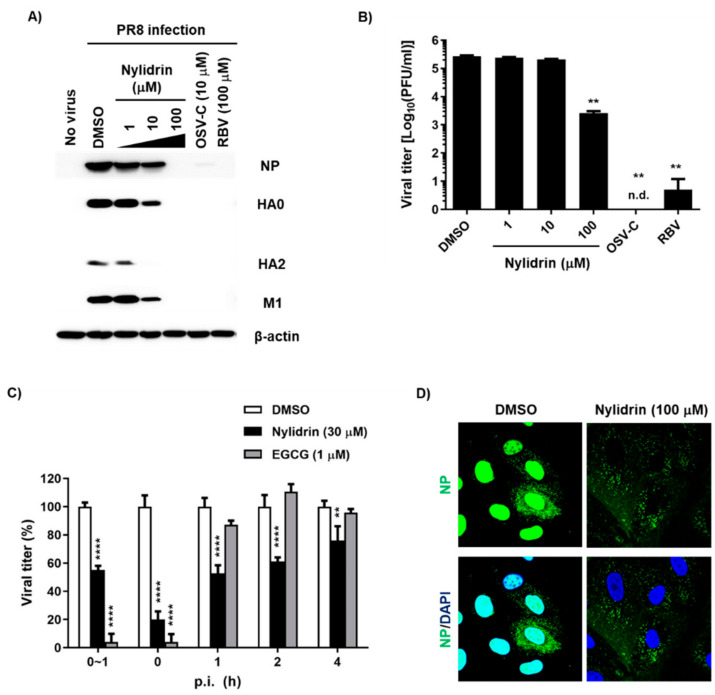
Inhibition of influenza virus infection by nylidrin. (**A**) Western blot analysis showing the dose-dependent decrease in viral proteins by nylidrin. Madin-Darby canine kidney (MDCK) cells were mock-infected (no virus) or infected for 24 h with the PR8 virus at a multiplicity of infection (MOI) of 0.001 in the presence of dimethyl sulfoxide (DMSO; 0.2%, a delivery vehicle), nylidrin (1, 10 or 100 μM), oseltamivir carboxylate (OSV-C; 10 μM), or ribavirin (RBV; 100 μM). Each viral protein and α-actin, as a loading control, are marked on the right side of the gels. (**B**) Plaque reduction assay. Viral titers were determined using culture supernatants from the same samples as in (A). This represents one of the three independent experiments. Statistical comparisons were performed using a two-tailed unpaired *t*-test. *n* = 2; **, *p* < 0.01. n.d., not detected. (**C**) Time-of-addition experiment. Nylidrin or (−)-epigallocatechin gallate (EGCG; an entry blocker) was administered during virus adsorption at 4 °C for 1 h (0~1 h) or at the indicated time points post-infection (p.i.) (0, 1, 2, and 4 h). At 5 h p.i., the supernatants were removed and replaced with an overlay medium for incubation at 35 °C. The percentage plaque number was determined by crystal violet staining on day 3. This represents one of the three independent experiments. Statistical significance was assessed using a two-way ANOVA with Dunnett’s multiple comparisons tests. *n* = 2; **, *p* < 0.01; ****, *p* < 0.0001. (**D**) Confocal microscopy. MDCK cells were infected with PR8 (MOI, 1) at 37 °C for 5 h in the presence of DMSO (delivery vehicle) or nylidrin (100 μM). Viral NP (green) and cellular nuclei (blue) were detected using NP-specific antibody and DAPI, respectively. Original magnification, 400×.

In addition to the results from the indirect CPE-based antiviral assay, it was necessary to determine whether nylidrin could inhibit influenza viral infection by monitoring viral proteins or plaque reduction directly. We treated PR8-infected MDCK cells with increasing concentrations of nylidrin in which OSV-C and RBV were used as inhibitory controls. Western blot analysis of cell lysates revealed a dose-dependent decrease in the levels of three viral proteins, NP, HA, and M1, consistently (Figure 2A). Notably, at the maximum concentration (100 μM), these proteins were not detected, as in the OSV-C- and RBV-treated samples. This finding was reproduced in A549 cells, indicating that nylidrin has antiviral activity in human lung cells, and not limited in a canine kidney-derived cell line (Supplementary Figure S2). Culture supernatants also exhibited a significant reduction in the viral plaque number in the presence of nylidrin (Figure 2B). These results demonstrate that nylidrin is an anti-influenza viral compound that downregulates viral protein expression and the production of infectious progeny virus.

To further investigate which step in the virus life cycle is targeted by nylidrin, we performed treatment during adsorption or at various time points p.i. over a total time of 5 h, in which EGCG was used as a control for the blocking of viral entry. This time-of-addition experiment revealed that nylidrin affected influenza virus replication in an incubation period-dependent manner (Figure 2C). Immunofluorescence imaging of viral NP at 5 h p.i. confirmed that nylidrin prevents the virus entry step, particularly after attachment or cellular membrane penetration of the virus, but before its RNA-dependent RNA replication in the nucleus, resulting in abnormal accumulation of vRNP in the cytoplasm (Figure 2D).

### 3.4. Inhibition of HA2 Fusion Activity by Nylidrin

**Figure 3:**
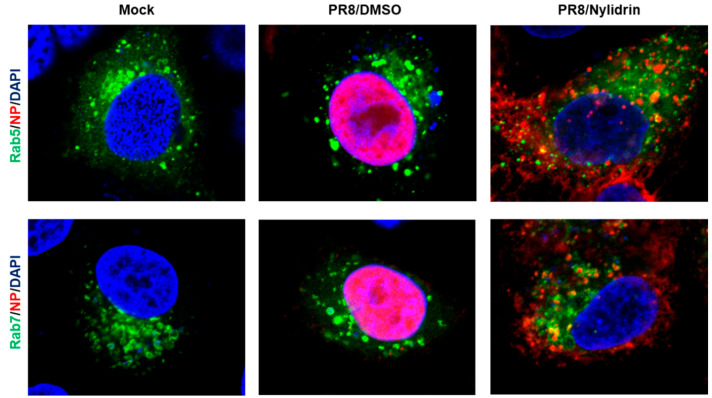
Colocalization of vRNP with endosomal makers, Rab5 and Rab7. A549 cells were transfected with pEGFP-Rab5 or -Rab7 expression plasmid. On the next day, PR8 virus mixed with DMSO or nylidrin (100 μM) were infected into A549 cells at an MOI of 10 at 4 °C for 30 min. After additional 4 h-incubation at 37 °C, cells were fixed and permeabilized for probing anti-NP antibody and Alexa 633-conjugated secondary antibody. Mock, no PR8 infection. Green, an EGFP-tagged endosomal marker, Rab5 (upper) or Rab7 (lower). Red, viral NP. Blue, cellular nuclei stained with DAPI. Original magnification, 630×.

Given the limitation of nuclear migration of vRNPs in the presence of nylidrin (Figure 2D), we hypothesized that the compound could target one of the three viral protein functions during the virus entry step, (1) M2 proton channel, (2) HA2 fusion protein, and (3) NP NLSs-mediated nuclear import of vRNPs. To determine which protein is involved in antiviral activity, we first examined whether the cytoplasmic vRNP complexes were internalized into endosomal compartments. Confocal microscopy clearly revealed their colocalization with an early endosome marker, Rab5, and more frequently with a late endosomal marker, Rab7, at the perinuclear region by nylidrin at 4.5 h p.i., when NP had completely migrated to the nucleus in the absence of nylidrin (Figure 3). This result suggested that nylidrin could block the escape of vRNPs from the endosomes, simply excluding their NP-mediated nuclear import as a target step.

**Figure 4:**
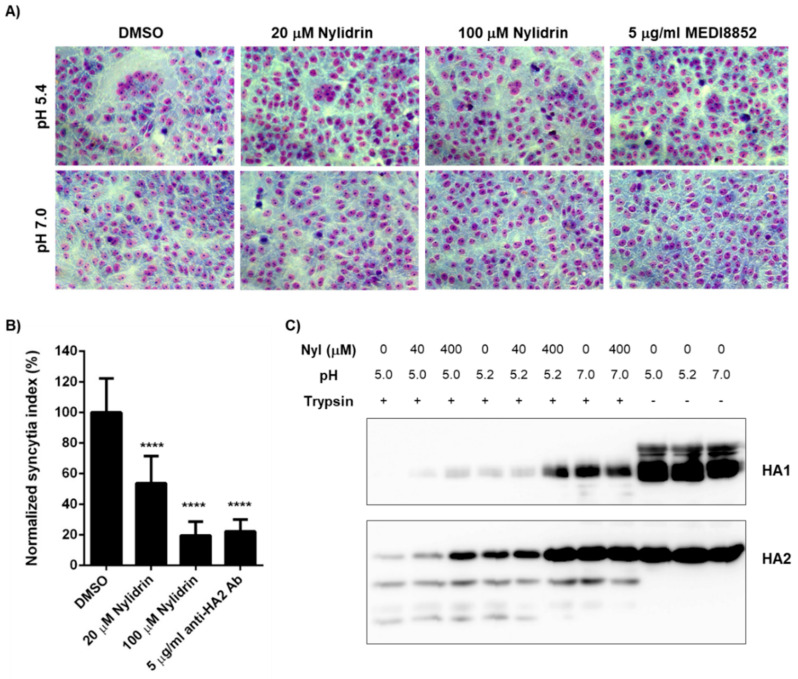
Inhibition of influenza HA2-mediated membrane fusion by nylidrin. (**A**) Polykaryon formation assay. PR8-infected Vero E6 cells (MOI, 1) were incubated under acidic (pH 5.4) or neutral (pH 7.0) conditions for 15 min at 37 °C in the presence of nylidrin (20 or 100 μM) or the anti-HA2 antibody MEDI8852 (5 μg/mL). After syncytium formation for additional 3 h at neutral pH, the cells were fixed and stained with Giemsa for microscopic analysis (200×). (**B**) Percentages of syncytium formation relative to the DMSO-treated control from 16 non-overlapping images; ****, *p* < 0.0001. Statistical comparisons were performed using a two-tailed unpaired *t*-test. (**C**) Tryptic digestion assay. PR8 virus (5.5 × 10^7^ PFU) was incubated with DMSO or nylidrin (40 or 400 μM) for 20 min. Under acidic (pH 5.0 or 5.2) or neutral (pH 7.0) conditions, the samples were individually incubated for 15 min. After neutralizing with Tris-HCl (pH 9.0), they were treated with (+) or without (-) trypsin for 1 h and subjected to western blot analysis to detect viral HA1 and HA2 proteins. Uncleaved proteins by trypsin are marked to the right of the panels.

To narrow down the mode-of-action study, we prepared retrovirus-derived virus-like particles (VLPs) spiked with M2. As the wild-type PR8 M2 (named PR8M2-R) is intrinsically resistant to AMT, we constructed the A27V/N31S reverse mutant (named PR8M2-S), which is sensitive to the antiviral drug, thereby generating PR8M2-S- and PR8M2-R-coupled VLPs [16]. Real-time proton channel activity assay revealed that nylidrin had no effect on the function of either PR8M2-R or PR8M2-S under an acidic condition (pH 5.2), while AMT inhibited PR8M2-S selectively at the same condition (Supplementary Figure S3). Therefore, we further investigated whether the compound could interfere with the rest function (i.e., HA2-mediated membrane fusion). Polykaryon formation assay revealed that syncytia formation by PR8-infected Vero E6 cells was suppressed by nylidrin as efficiently as by MEDI8852, a neutralizing antibody bound to HA2 (Figure 4A). In quantitative terms, 80.5% and 77.8% of viral HA2-mediated polykaryon formation was inhibited by 100 μM nylidrin and 5 μg/mL MEDI8852, respectively (Figure 4B). We then examined whether nylidrin prevents the low pH-induced conformational change in HA. The tryptic digestion assay showed that nylidrin affects the sensitivity of both HA1 and HA2 to trypsin under acidic conditions (both pH 5.0 and 5.2), but not under a neutral condition (pH 7.0) (Figure 4C). Taken together, the results strongly suggest that nylidrin targets HA2-mediated membrane fusion by preventing the whole HA protein from undergoing conformational changes at low pH.

### 3.5. In Vivo Antiviral Efficacy of Nylidrin 

**Figure 5:**
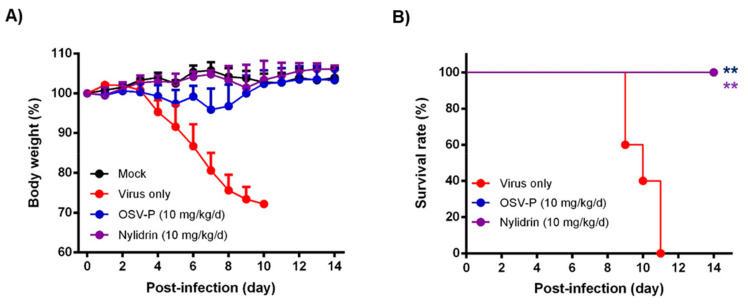
In vivo antiviral activity of nylidrin against a mouse-adapted influenza A virus (H1N1), maPR8. Before viral challenge, maPR8 (5 MLD_50_) was preincubated with DMSO (virus only) or with nylidrin (10 mg/kg) at room temperature for 30 min. Mice were mock-infected or infected with the preincubated samples. As a positive control, OSV-P (10 mg/kg/day) was orally administered twice a day beginning 4 h before virus infection at 8-h intervals from days 0 to 5 post-infection. Body weight (**A**) and mortality (**B**) were measured every day for 15 days. Statistical analysis was performed using a two-tailed unpaired *t*-test relative to the virus-only group. *n* = 5; **, *p* < 0.01.

Next, it was studied whether nylidrin, as an HA2-mediated fusion inhibitor, could protect mice from viral infection. To test its ability, we intranasally infected immunocompetent BALB/c mice with 5 MLD_50_ of maPR8 following preincubation of the virus with DMSO (delivery vehicle) or nylidrin (10 mg/kg) at room temperature for 30 min (Figure 5). As a therapeutic control, maPR8-infected mice were orally administered with OSV-P twice per day at a dose of 10 mg/kg/day from days 0 to 5 p.i. beginning 4 h before virus challenge. Body weight changes and survival rates confirmed that nylidrin not only mitigated body weight loss induced by viral infection (Figure 5A), but also prevented mortality: complete survival (100%) was comparable to that in the OSV-P-treated group (Figure 5B). In contrast, mice infected with the vehicle-treated maPR8 died between days 9 and 11. Taken together, these findings suggest that nylidrin protects mice from viral infection in vivo by directly targeting the HA2 function of influenza A virus (H1N1).

## 4. Discussion

From screening of a chemical library, we identified nylidrin, an ADRB2 agonist, as an antiviral compound against influenza A virus. ADRB2 is a G protein-coupled receptor (GPCR) and primarily expressed on smooth muscle cells surrounding the bronchioles. ADRB2 exists in equilibrium between activated and inactivated forms under resting conditions. Agonists trigger its activation, resulting in conversion of adenosine triphosphate (ATP) into cyclic AMP (cAMP) by adenylate cyclase in a G_s_-type G protein-dependent manner [26]. Subsequent sequestration of intracellular Ca^2+^ leads to muscle relaxation [27]. Related to the roles of adrenergic receptors on viral infection, it has been suggested that ADRB2 agonists could downregulate the innate immune response and reduce host resistance to viral infection in a murine cytomegalovirus infection model [28]. Another study reported that clonidine, an agonist of the ⍺2 adrenergic receptor (ARDA2), inhibited various influenza strains at the viral assembly step [29]. As a counterplayer of ADRB2, ARDA2, a G_i_-type G protein-coupled receptor, decreases intracellular cAMP levels when bound to an agonistic molecule. On the basis of these two previous reports, it was wondered how ADRB2 agonists have antiviral activity against the influenza A virus. Here, this question was answered by the 3D-QSAR analysis and by better understanding of the mechanism of antiviral action. Primarily, in phenotypic antiviral assays, not only nylidrin, but also other GPCR-regulatory molecules including ifenprodil and clenbuterol exhibited antiviral activity against A/H1N1 and/or A/H3N2 strains with varying degrees of efficacy. However, not all ADRB2 agonists suppressed the virus infection (e.g., ritodrine, fenoterol, and bambuterol) were inactive. This inspired us to try a field-based pharmacophore analysis of the chemical analogues by fixing the phenyl aminoethanol motif as a core skeleton (Figure 1) and explained why particularly ifenprodil and clenbuterol were as potent as nylidrin. It was because they shared a highly similar electrostatic potential map at the molecular level. No relationship between antiviral activity and ADRB2 agonistic function implied that nylidrin could be a direct-acting antiviral agent, instead of an indirectly acting, host factor regulatory one.

Decisively, the polykaryon formation assay verified that nylidrin targets the HA2-mediated membrane fusion by blocking pH-dependent conformational change of HA. As above-mentioned, additional evidence supporting this hypothesis was provided by subtype dependency on the antiviral efficacy: nylidrin, ifenprodil, and clenbuterol were active against all A/H1N1 strains tested; in contrast, nylidrin and ifenprodil were limitedly effective only against A/Hong Kong/8/1968 and A/Seoul/11/1988 among the H3N2 strains tested (Table 1 and Table 2). This observation is intriguing because most HA2 fusion inhibitory agents, either therapeutic antibodies [30,31,32] or small molecules [22,33,34], tend to exhibit antiviral potency in a viral subtype- or HA group-dependent manner; group I consists of H1, H2, and H5, whereas group 2 consists of H3 and H7. The strain-specific activity of the compounds against the H3N2 subtype viruses indicated that optimization of nylidrin through chemical modifications could be a plausible approach for identifying a broad-spectrum fusion inhibitor.

Another point to be stressed is that nylidrin completely blocked intranasal influenza infections of mice when it was preincubated with the virus (Figure 5). Even though the OSV-P–treated group showed a complete therapeutic effect, it led to transient decreases in body weight after virus challenge. Meanwhile, the nylidrin–treated group exhibited an overall body weight increase without mortality during the monitoring period, nearly identical to the mock–infected group. In light of this result, we examined the efficacy of the oral administration of nylidrin twice a day (at a dose of 200 mg/kg/day) for 13 days p.i. Unfortunately, oral post-treatment did not improve infection-mediated mortality, but instead moderated the slope of body weight loss relative to the virus-only group, enhancing mean survival time by 1.4 days (Supplementary Figure S4). These observations suggest that although nylidrin is a potent, direct-acting inhibitor of influenza virus HA2, the pharmacokinetics of gastrointestinal absorption is not sufficient for oral administration to treat influenza virus infection. Future studies could also focus on chemical modifications that would render a broader range of viral strains sensitive to nylidrin derivatives as well as improve the pharmacokinetic properties for their therapeutic use.

## Figures and Tables

**Table 1:** Antiviral activity of nylidrin and its analogues against influenza viruses, PR8, HK, and Lee.

Compound	CC_50_ (μM) ^a^ to MDCK Cells	EC_50_ (μM) ^b^ against Influenza Viruses (S.I. ^c^)			Function
		A/ Puerto Rico/8/1934 (H1N1)	A/Hong Kong/8/1968 (H3N2)	B/Lee/40	
Nylidrin	549.2 ± 0.5	7.2 ± 0.2	12.1 ± 1.6	>100.0	β2-Adrenergic receptor agonist
		(76.3)	(45.4)	(N.D.)	
Ifenprodil	488.2 ± 0.5	6.6 ± 0.4	16.9 ± 3.0	>100.0	N-methyl-_D_-aspartate receptor 2B antagonist
		(74.0)	(29.0)	(N.D. ^d^)	
Labetalol	498.1 ± 0.5	>100.0	44.0 ± 9.7	>100.0	α1- and β1/β2-Adrenergic receptor antagonist
		(N.D.)	(>11.3)	(N.D.)	
Ritodrine	>900.0	>100.0	>100.0	>100.0	β2-Adrenergic receptor agonist
		(N.D.)	(N.D.)	(N.D.)	
Fenoterol	>900.0	>100.0	>100.0	>100.0	β2-Adrenergic receptor agonist
		(N.D.)	(N.D.)	(N.D.)	
Eliprodil	>900.0	>100.0	58.1 ± 6.4	>100.0	N-methyl-_D_-aspartate antagonist
		(N.D.)	(>15.5)	(N.D.)	
Clenbuterol	>900.0	9.4 ± 4.6	>100.0	>100.0	β2-Adrenergic receptor agonist
		(>96.3)	(N.D.)	(N.D.)	
Bambuterol	>900.0	>100.0	>100.0	>100.0	β2-Adrenergic receptor agonist
		(N.D.)	(N.D.)	(N.D.)	
AMT^e^	>900.0	>100.0	0.8 ± 0.1	>100.0	- ^f^
		(N.D.)	(>1200)	(N.D.)	
RBV ^g^	>900.0	18.4 ± 3.4	15.7 ± 2.7	13.8 ± 0.2	-
		(>48.9)	(>57.5)	(>65.5)	
OSV-C ^h^	>900.0	0.04 ± 0.01	<0.005	0.8 ± 0.03	-
		(>25,714)	(>180,000)	(>1125)	

^a^ 50% cytotoxic concentration (i.e., the concentration at which cell viability was reduced by 50%); ^b^ 50% effective concentration [i.e., the concentration required to improve viability of influenza-infected Madin-Darby canine kidney (MDCK) cells by 50%]; ^c^ Selectivity index (ratio of CC_50_/EC_50_); ^d^ not determined; ^e^ amantadine hydrochloride; ^f^ control compound; ^g^ ribavirin; ^h^ oseltamivir carboxylate.

**Table 2:** Antiviral activity of nylidrin and its analogues against various influenza A and B viruses.

Compound	CC_50_ (μM) ^a^ to MDCK Cells	EC_50_ (μM) ^b^ against Influenza Viruses (S.I. ^c^)						
		A/California /7/2009 (H1N1)	A/Brisbane /59/2007 (H1N1)	A/Seoul /11/1988 (H3N2)	A/Perth /16/2009 (H3N2)	A/Brisbane /10/2007 (H3N2)	A/Victoria /361/2011-like (H3N2)	B/Brisbane /60/2008
Nylidrin	549.2 ± 0.5	1.7 ± 0.5	3.5 ± 1.6	38.8 ± 19.4	>100.0	>100.0	>100.0	>100.0
		(332.8)	(156.9)	(14.3)	(N.D. ^d^)	(N.D.)	(N.D.)	(N.D.)
Ifenprodil	488.2 ± 0.5	1.1 ± 0.1	5.1 ± 0.3	37.9 ± 7.7	>100.0	>100.0	>100.0	>100.0
		(465.0)	(96.7)	(14.5)	(N.D.)	(N.D.)	(N.D.)	(N.D.)
Labetalol	498.1 ± 0.5	>100.0	>100.0	>100.0	>100.0	>100.0	>100.0	>100.0
		(N.D.)	(N.D.)	(N.D.)	(N.D.)	(N.D.)	(N.D.)	(N.D.)
Eliprodil	>900.0	>100.0	53.1 ± 0.3	>100.0	>100.0	>100.0	>100.0	>100.0
		(N.D.)	(>16.9)	(N.D.)	(N.D.)	(N.D.)	(N.D.)	(N.D.)
Clenbuterol	>900.0	13.7 ± 3.6	12.9 ± 0.3	>100.0	>100.0	>100.0	>100.0	>100.0
		(>65.9)	(>69.8)	(N.D.)	(N.D.)	(N.D.)	(N.D.)	(N.D.)
AMT ^e^	>900.0	>100.0	1.2 ± 0.4	0.3 ± 0.1	>100.0	>100.0	>100.0	>100.0
		(N.D.)	(>750)	(>1831)	(N.D.)	(N.D.)	(N.D.)	(N.D.)
RBV ^f^	>900.0	52.5 ± 1.5	24.1 ± 0.5	10.5 ± 4.2	33.8 ± 11.6	12.6 ± 6.4	33.4 ± 8.8	25.6 ± 0.3
		(>17.2)	(>37.3)	(>52.3)	(>26.6)	(>71.4)	(>16.4)	(>35.2)
OSV-C ^g^	>900.0	0.02 ± 0.0	0.02 ± 0.0	<0.005	0.03 ± 0.02	0.53 ± 0.16	<0.005	53.1 ± 25.25
		(>58,065)	(>58,065)	(>180,000)	(>30,000)	(>1714)	(>180,000)	(>17.0)

^a^ 50% cytotoxic concentration (i.e., the concentration at which cell viability was reduced by 50%); ^b^ 50% effective concentration (i.e., the concentration required to improve viability of influenza-infected MDCK cells by 50%); ^c^ Selectivity index (ratio of CC_50_/EC_50_); ^d^ not determined; ^e^ amantadine hydrochloride; ^f^ ribavirin; ^g^ oseltamivir carboxylate.

## Supplementary Materials

The following are available online at https://www.mdpi.com/1999-4915/12/5/581/s1, Figure S1: Chemical structure of nylidrin and its analogues, Figure S2: Reduction of a viral protein, nucleoprotein (NP) by nylidrin in human lung epithelial cells, Figure S3: No effect of nylidin on the proton channel activity of influenza viral M2, Figure S4: Increase of mean survival time of maPR8-infected mice by oral administration of nylidrin.

## References

[1] Arranz R., Coloma R., Chichon F.J., Conesa J.J., Carrascosa J.L., Valpuesta J.M., Ortin J., Martin-Benito J. (2012). The structure of native influenza virion ribonucleoproteins. Science.

[2] Pflug A., Guilligay D., Reich S., Cusack S. (2014). Structure of influenza A polymerase bound to the viral RNA promoter. Nature.

[3] Nakatsu S., Sagara H., Sakai-Tagawa Y., Sugaya N., Noda T., Kawaoka Y. (2016). Complete and Incomplete Genome Packaging of Influenza A and B Viruses. mBio.

[4] Weis W., Brown J.H., Cusack S., Paulson J.C., Skehel J.J., Wiley D.C. (1988). Structure of the influenza virus haemagglutinin complexed with its receptor, sialic acid. Nature.

[5] Gamblin S.J., Skehel J.J. (2010). Influenza hemagglutinin and neuraminidase membrane glycoproteins. J. Biol. Chem..

[6] Bullough P.A., Hughson F.M., Skehel J.J., Wiley D.C. (1994). Structure of influenza haemagglutinin at the pH of membrane fusion. Nature.

[7] Pinto L.H., Lamb R.A. (2006). The M2 proton channels of influenza A and B viruses. J. Biol. Chem..

[8] Neumann G., Castrucci M.R., Kawaoka Y. (1997). Nuclear import and export of influenza virus nucleoprotein. J. Virol..

[9] Abraham G.M., Morton J.B., Saravolatz L.D. (2020). Baloxavir: A Novel Antiviral Agent in the Treatment of Influenza. Clin. Infect. Dis..

[10] De Clercq E. (2006). Antiviral agents active against influenza A viruses. Nat. Rev. Drug Discov..

[11] Bragstad K., Hungnes O., Litleskare I., Nyrerod H.C., Dorenberg D.H., Hauge S.H. (2019). Community spread and late season increased incidence of oseltamivir-resistant influenza A(H1N1) viruses in Norway 2016. Influenza Other Respir. Viruses.

[12] Pielak R.M., Schnell J.R., Chou J.J. (2009). Mechanism of drug inhibition and drug resistance of influenza A M2 channel. Proc. Natl. Acad. Sci. USA.

[13] Hurt A.C., Besselaar T.G., Daniels R.S., Ermetal B., Fry A., Gubareva L., Huang W., Lackenby A., Lee R.T., Lo J. (2016). Global update on the susceptibility of human influenza viruses to neuraminidase inhibitors, 2014-2015. Antiviral Res..

[14] Hurt A.C., Chotpitayasunondh T., Cox N.J., Daniels R., Fry A.M., Gubareva L.V., Hayden F.G., Hui D.S., Hungnes O., Lackenby A. (2012). Antiviral resistance during the 2009 influenza A H1N1 pandemic: Public health, laboratory, and clinical perspectives. Lancet Infect. Dis..

[15] Checkmahomed L., M’Hamdi Z., Carbonneau J., Venable M.C., Baz M., Abed Y., Boivin G. (2020). Impact of the Baloxavir-Resistant Polymerase Acid I38T Substitution on the Fitness of Contemporary Influenza A(H1N1) pdm09 and A(H3N2) Strains. J. Infect. Dis..

[16] Jang Y., Shin J.S., Yoon Y.S., Go Y.Y., Lee H.W., Kwon O.S., Park S., Park M.S., Kim M. (2018). Salinomycin Inhibits Influenza Virus Infection by Disrupting Endosomal Acidification and Viral Matrix Protein 2 Function. J. Virol..

[17] Kim M., Kim S.Y., Lee H.W., Shin J.S., Kim P., Jung Y.S., Jeong H.S., Hyun J.K., Lee C.K. (2013). Inhibition of influenza virus internalization by (-)-epigallocatechin-3-gallate. Antiviral Res..

[18] Niemeyer G., Cottier D., Resch H. (1987). Effects of buphenine (nylidrin) on the perfused mammalian eye. Graefes Arch. Clin. Exp. Ophthalmol..

[19] Shin J.S., Ku K.B., Jang Y., Yoon Y.S., Shin D., Kwon O.S., Go Y.Y., Kim S.S., Bae M.A., Kim M. (2017). Comparison of anti-influenza virus activity and pharmacokinetics of oseltamivir free base and oseltamivir phosphate. J. Microbiol..

[20] Jang Y., Lee H.W., Shin J.S., Go Y.Y., Kim C., Shin D., Malpani Y., Han S.B., Jung Y.S., Kim M. (2016). Antiviral activity of KR-23502 targeting nuclear export of influenza B virus ribonucleoproteins. Antiviral Res..

[21] Vinter J.G. (1994). Extended electron distributions applied to the molecular mechanics of some intermolecular interactions. J. Comput Aided Mol Des..

[22] Vanderlinden E., Goktas F., Cesur Z., Froeyen M., Reed M.L., Russell C.J., Cesur N., Naesens L. (2010). Novel inhibitors of influenza virus fusion: Structure-activity relationship and interaction with the viral hemagglutinin. J. Virol..

[23] Kallewaard N.L., Corti D., Collins P.J., Neu U., McAuliffe J.M., Benjamin E., Wachter-Rosati L., Palmer-Hill F.J., Yuan A.Q., Walker P.A. (2016). Structure and Function Analysis of an Antibody Recognizing All Influenza A Subtypes. Cell.

[24] Yoon H., Song J.M., Ryu C.J., Kim Y.G., Lee E.K., Kang S., Kim S.J. (2012). An efficient strategy for cell-based antibody library selection using an integrated vector system. BMC Biotechnol..

[25] Mittag T.W., Tormay A., Messenger M., Podos S.M. (1985). Ocular hypotension in the rabbit. Receptor mechanisms of pirbuterol and nylidrin. Invest. Ophthalmol. Vis. Sci..

[26] Liggett S.B. (2002). Update on current concepts of the molecular basis of beta2-adrenergic receptor signaling. J. Allergy Clin. Immunol..

[27] Johnson M. (2006). Molecular mechanisms of beta (2)-adrenergic receptor function, response, and regulation. J. Allergy Clin. Immunol..

[28] Wieduwild E., Girard-Madoux M.J., Quatrini L., Laprie C., Chasson L., Rossignol R., Bernat C., Guia S., Ugolini S. (2020). beta2-adrenergic signals downregulate the innate immune response and reduce host resistance to viral infection. J. Exp. Med..

[29] Matsui K., Ozawa M., Kiso M., Yamashita M., Maekawa T., Kubota M., Sugano S., Kawaoka Y. (2018). Stimulation of alpha2-adrenergic receptors impairs influenza virus infection. Sci. Rep..

[30] Li T.W., Cheng S.F., Tseng Y.T., Yang Y.C., Liu W.C., Wang S.C., Chou M.J., Lin Y.J., Wang Y., Hsiao P.W. (2016). Development of single-chain variable fragments (scFv) against influenza virus targeting hemagglutinin subunit 2 (HA2). Arch. Virol..

[31] Gocnik M., Fislova T., Sladkova T., Mucha V., Kostolansky F., Vareckova E. (2007). Antibodies specific to the HA2 glycopolypeptide of influenza A virus haemagglutinin with fusion-inhibition activity contribute to the protection of mice against lethal infection. J. Gen. Virol..

[32] Prabhu N., Prabakaran M., Ho H.T., Velumani S., Qiang J., Goutama M., Kwang J. (2009). Monoclonal antibodies against the fusion peptide of hemagglutinin protect mice from lethal influenza A virus H5N1 infection. J. Virol..

[33] Basu A., Antanasijevic A., Wang M., Li B., Mills D.M., Ames J.A., Nash P.J., Williams J.D., Peet N.P., Moir D.T. (2014). New small molecule entry inhibitors targeting hemagglutinin-mediated influenza a virus fusion. J. Virol..

[34] White K.M., De Jesus P., Chen Z., Abreu P., Barile E., Mak P.A., Anderson P., Nguyen Q.T., Inoue A., Stertz S. (2015). A Potent Anti-influenza Compound Blocks Fusion through Stabilization of the Prefusion Conformation of the Hemagglutinin Protein. ACS Infect. Dis..

