# Supplementary material for: In Vitro and In Vivo Antiviral Activity of Nylidrin by Targeting the Hemagglutinin 2-Mediated Membrane Fusion of Influenza A Virus
Source: Viruses. 2020 May 25;12(5):581. doi: 10.3390/v12050581 (PMC7290441; doi:10.3390/v12050581)
Supplement: Supplementary file 1 [file viruses-12-00581-s001.pdf]

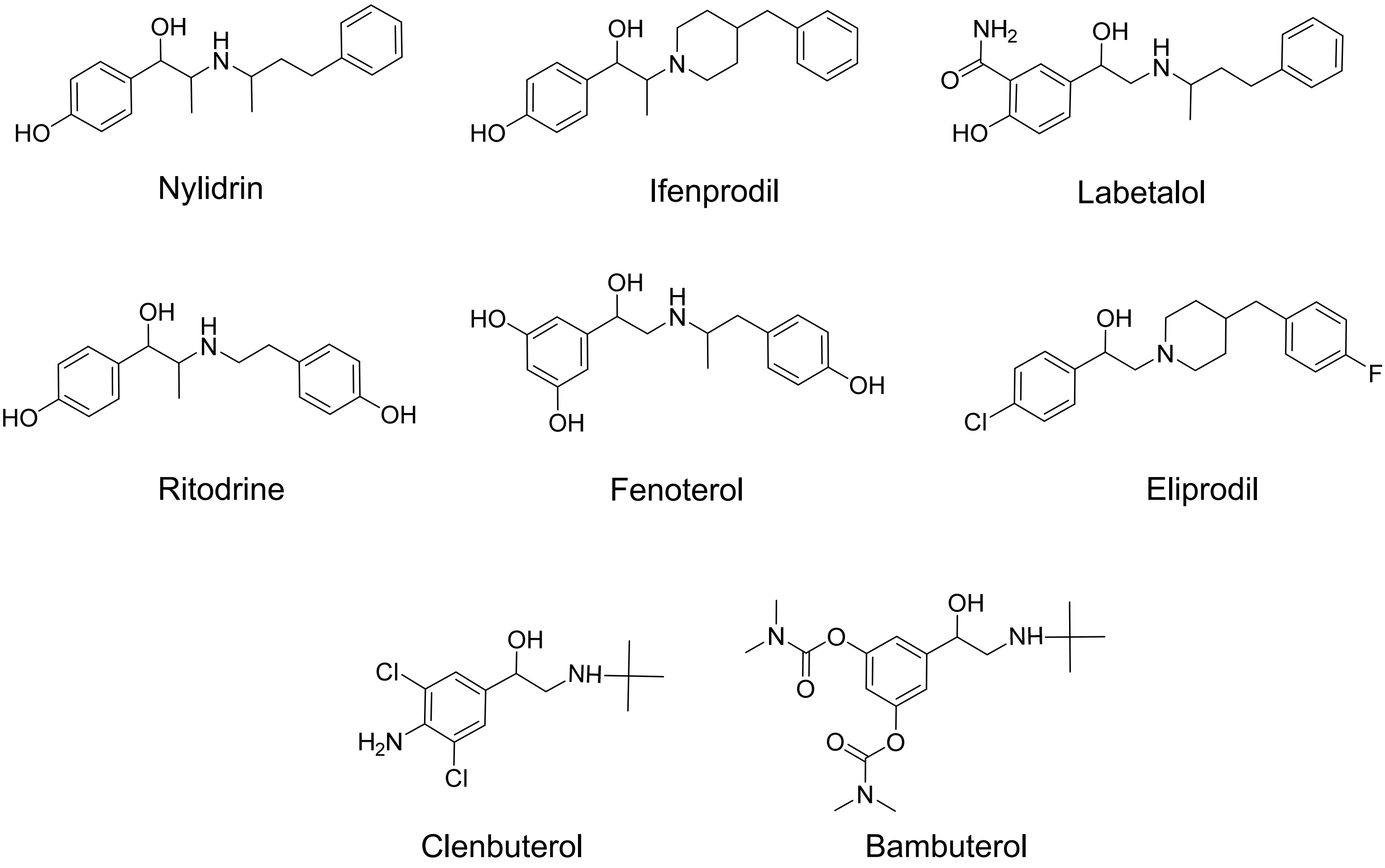

Supplementary Figure S1. Chemical structure of nylidrin and its analogues.

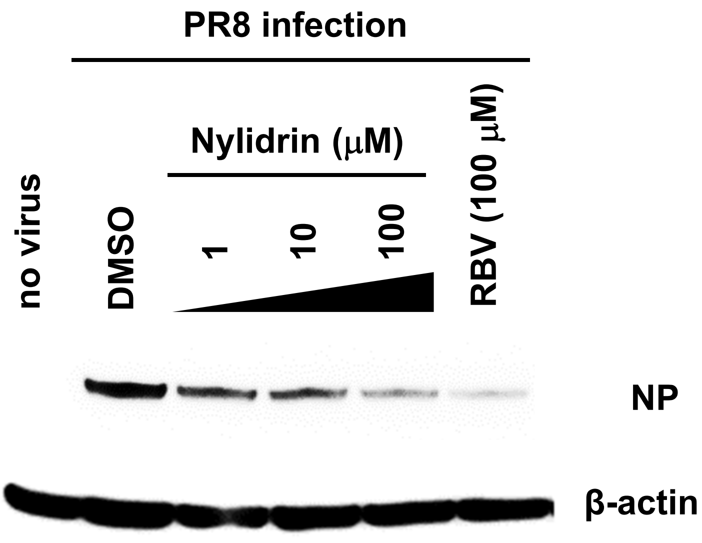

Supplementary Figure S2. Reduction of a viral protein, NP, by nylidrin in human lung epithelial cells. A549 cells were mock-infected (No virus) or infected with PR8 virus at an MOI of 0.01 in the presence of DMSO (0.2%, a delivery vehicle), nylidrin (1, 10 or 100 μM) or RBV (100 μM) for 24 h at 35°C. NP and β-actin, as a loading control, are marked on the right side of the gels.

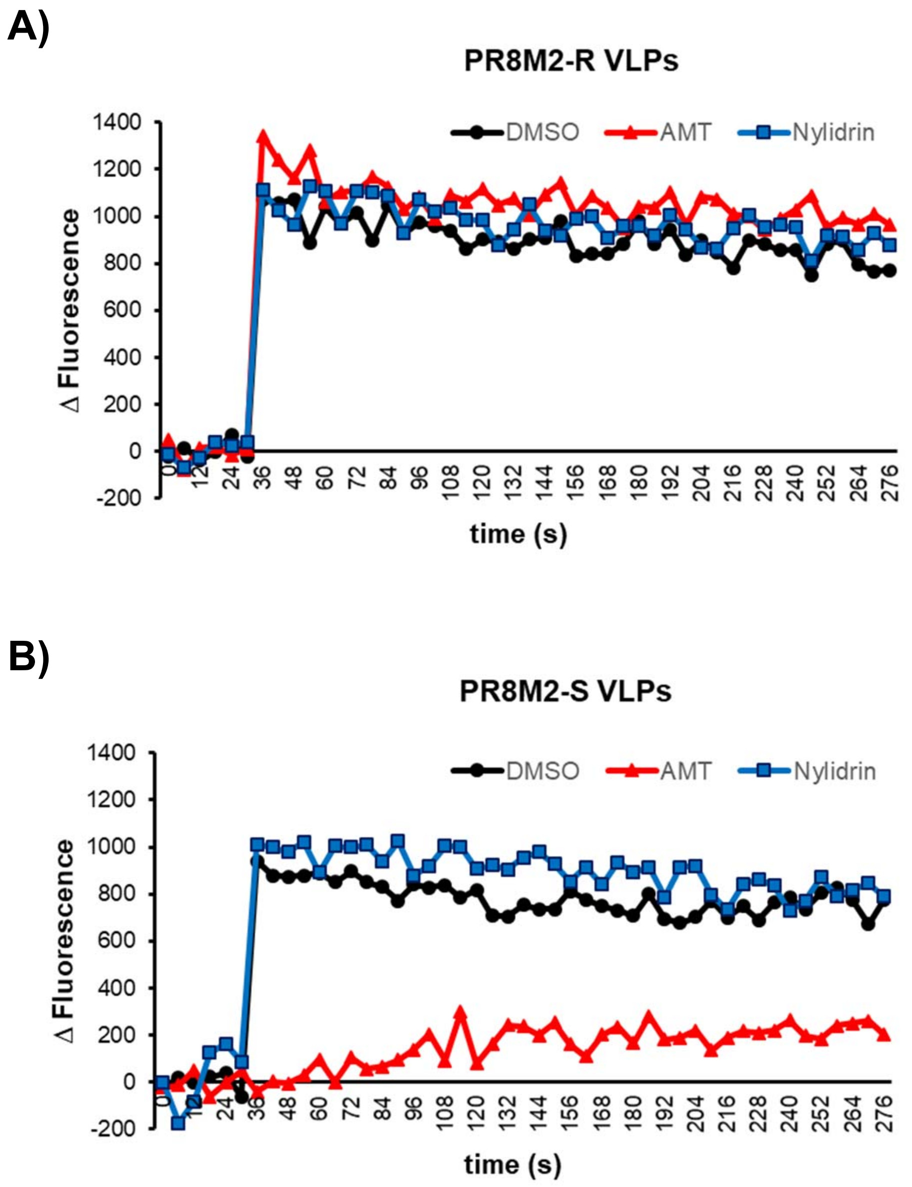

Supplementary Figure S3. No effect of nylidin on the proton channel activity of influenza viral M2. Murine leukemia virus (MLV) Gag-derived VLPs with PR8M2-R (A) or PR8M2-S (B) were suspended in 10 mM HEPES (pH 7.0) and 150 mM NaCl supplemented with 1% FMP-Blue dye (Molecular Deivces, Sunnyvale, CA). They were incubated with 100 μM salinomycin or AMT at room temperature for 1 h. Channel activity was measured at 6 s intervals for 5 min after addition of 150 mM 2-(N-morpholino)ethanesulfonic acid (pH 4.5). DMSO-treated VLPs were used as a control. Proton channel activity was determined by measuring fluorescence at 6 s intervals for 5 min. Values represent the average of three independent experiments.

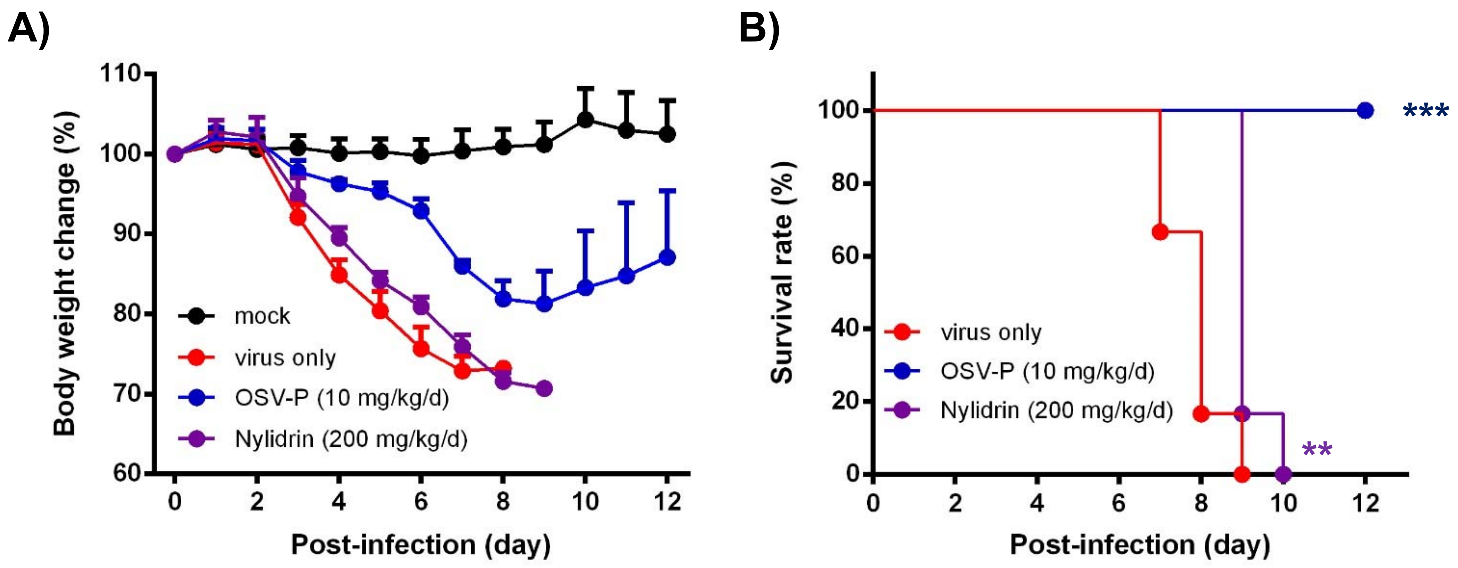

Supplementary Figure S4. Increase of mean survival time of maPR8-infected mice by oral administration of nylidrin. BALB/c mice (6–7 weeks old) infected with maPR8 (5 MLD_{50}) were orally treated with OSV-P (10 mg/kg/day) or nylidrin (200 mg/kg/day) twice a day beginning 4 h before virus infection at 8-h intervals for 6 days or 13 days post-infection, respectively. Body weight (A) and mortality (B) were measured every day. Statistical analysis was performed using the two-tailed Student's *t*-test relative to the virus-only group. $n = 6$; **, $P < 0.01$; ***, $P < 0.001$.
